# Satisfaction and experiences of patients taking fingolimod and involved in a pharmacy-based patient support program in Switzerland — a qualitative study

**DOI:** 10.1186/s12913-020-05278-3

**Published:** 2020-05-14

**Authors:** Aline Bourdin, Julie Dubois, Rose-Anna Foley, Myriam Schluep, Olivier Bugnon, Jérôme Berger

**Affiliations:** 1 Community Pharmacy, Center for Primary Care and Public Health (Unisanté), University of Lausanne, Lausanne, Switzerland; 2 School of Pharmaceutical Sciences, University of Geneva, Lausanne, Switzerland; 3 Institute of Pharmaceutical Sciences of Western Switzerland, University of Geneva, University of Lausannee, Lausanne, Switzerland; 4 Qualitative Research Platform, Center for Primary Care and Public Health (Unisanté), University of Lausanne, Lausanne, Switzerland; 5 Division of Neurology, Department of Clinical Neurosciences, Lausanne University Hospital, CHUV, Lausanne, Lausanne, Switzerland

**Keywords:** Fingolimod, Multiple sclerosis, Patient support program, Specialty pharmacy service, Patient perceptions, Patient satisfaction, Qualitative research

## Abstract

**Background:**

Fingolimod is an oral multiple sclerosis drug that is considered a specialty drug due to its high cost and safety issues. The Fingolimod Patient Support Program (F-PSP) is a specialty pharmacy service developed to ensure the responsible use of fingolimod by promoting patient safety and medication adherence. This study aims to explore the satisfaction, experiences and perceptions regarding the F-PSP among patients currently involved in this program or recently withdrawn.

**Methods:**

A qualitative study was conducted via individual, face-to-face semistructured interviews with patients involved in the F-PSP. The interviews were audio-recorded, transcribed verbatim, coded and analyzed via thematic content analysis.

**Results:**

The main themes identified from the interviews (*n* = 17) were overall perception of the F-PSP, perception of the pharmacist-led consultations, perception of the tools (electronic monitor and drug intake graph), reasons to participate or potentially withdraw, and suggestions for improvements. Participants perceived the F-PSP as a reassuring support that complemented their medical care, providing a more human, personalized and person-centered approach than usual pharmacy care. Pharmacist-led consultations were valued for the medication-related and holistic support they provided. The importance of the pharmacist’s attitude was emphasized. The electronic monitor was valued for promoting daily medication adherence and allowing the involvement of relatives, which reassured participants and their relatives. The participants appreciated the drug intake graph because it provided an objective overview of medication adherence, thereby reassuring, rewarding, and motivating them. The main reason to join the program was to be supported, especially with respect to medication adherence.

**Conclusions:**

Participants were satisfied with the F-PSP, each for different reasons. Their feedback enabled the identification of measures for the optimization of the F-PSP and should facilitate its dissemination and transfer to other drugs/diseases/populations. Essential elements of generic pharmacist-led patient support programs considered valuable from the patients’ perspective were identified.

## Background

“Specialty drugs” are often defined as high-cost, high-complexity and/or high-touch medications that may require specific patient monitoring and management [1–3]. They are usually used to treat complex chronic conditions, such as multiple sclerosis (MS). Fingolimod, the first oral MS drug, is considered a specialty drug due to its high cost [4] and safety issues (cardiovascular, ophthalmic, hematologic, hepatic or pulmonary complications and risk of infections, cancer and fetal toxicity) [5]. Six hours of medical monitoring for bradycardia is required following the administration of the first dose, and various other medical tests are required prior to and after the initiation of fingolimod. Patients should be cautioned with regard to symptoms of potential serious adverse fingolimod reactions, and women with childbearing potential should be informed about the teratogenic risk and the use of effective contraception [6]. Moreover, lifelong drug intake is known to be challenging [7], and high medication adherence for MS treatment is associated with a decreased risk of relapses and medical costs [8, 9]. Therefore, supporting and monitoring patients taking fingolimod is recommended [6, 8]. In this context, the Community Pharmacy of the Center for Primary Care and Public Health (Unisanté), University of Lausanne (Switzerland), launched a specialty pharmacy service in 2013: the Fingolimod Patient Support Program (F-PSP).

The F-PSP aims to ensure responsible use of fingolimod and patient empowerment by promoting medication adherence and patient safety through a comprehensive and person-centered approach. The F-PSP has already been described in detail elsewhere [10]. It consists of face-to-face pharmacist-led consultations based on motivational interviewing techniques that follow a standardized timeline: monthly for the first 3 months and then quarterly (adaptable to patients’ needs). The pharmacists are supported by a secure web platform including a clinical decision aid system, patient health records and a data collection system. Patient safety is ensured through pharmacovigilance activity; the pharmacists provide fingolimod recommendations to the patients, remind them about recommended medical tests, and track their reported symptoms. Medication adherence is monitored and supported through an electronic monitor (EM) (MEMS®SmartCap, Aardex Group) in which fingolimod is repacked. The EM records and displays each opening on the cap screen, helping patients with their daily drug intake. A drug intake graph is uploaded, shown to patients and discussed during consultations. The intervention is based on the information-motivation-behavioral skills model [11], psychosocial needs and other moderating factors specific to each patient. At the end of the consultation, the pharmacist’s report is made available to the patient and the patient’s neurologist, MS nurse, general practitioner (GP), and/or other pharmacists to ensure continuity of care.

The F-PSP includes keys elements known to enhance medication adherence, such as EM feedback, cognitive-educational interventions providing patients with knowledge, counseling and accountability [12–14], as well as using motivational interviewing techniques [15, 16]. Additionally, delivering combined interventions seems to be the most effective strategy because multiple determinants influence medication adherence [17, 18]. The pharmacist appears as a key actor for effective pharmacovigilance programs and to support medication adherence [19, 20]. In addition, patients seem to have high level of satisfaction toward pharmacist-led services [21, 22].

The F-PSP is disseminated throughout the French speaking parts of Switzerland through a trained pharmacy network (22 pharmacies, including the Pharmacy of Unisanté). Participation in the F-PSP is proposed to each patient starting fingolimod at the MS clinic of the local university hospital and patients can join through any network pharmacy. Since its launch in 2013 to 2017, 70 patients have joined the F-PSP (58.3% of patients to whom it was proposed), including 52 patients at the Pharmacy of Unisanté. There are several reasons why patients may have chosen this particular pharmacy: it is located at the hospital site where the MS clinic is located, facilitating the pairing of pharmacist consultations with neurologist appointments. Moreover, the pharmacist presenting the program works in this pharmacy; thus, patients’ first contact with the program is through this pharmacist. Participation is voluntary, and patients can withdraw from the program at any time if they no longer feel it is necessary for them. In 2017, 34 patients were still participating (the median retention times of the 34 patients still participating and of the 36 who had withdrawn were 2.6 and 1.1 years, respectively).

Identification of the F-PSP’s strengths and weaknesses is needed to optimize the F-PSP (so as to retain the valued elements and change the unvalued ones), support its dissemination, transfer it to other specialty drugs/diseases/populations/healthcare contexts, and ultimately enhance patient care. The aim of this study was to explore the satisfaction, experiences and perceptions regarding the F-PSP among patients currently involved in this program or recently withdrawn.

## Methods

A qualitative study with a descriptive approach [23] was conducted via individual, face-to-face semistructured interviews. The purpose of this approach was to understand the meaning given by patients to the F-PSP and their perceptions and opinions regarding its usefulness.

### Participants

The participants were MS patients who initiated fingolimod treatment at the MS clinic and participated in the F-PSP. Patients were excluded if they were in the F-PSP for less than 3 months, withdrew from the F-PSP more than 6 months ago, withdrew from the F-PSP on the recommendation of a neurologist, or if a translator was required.

**Fig. 1:**
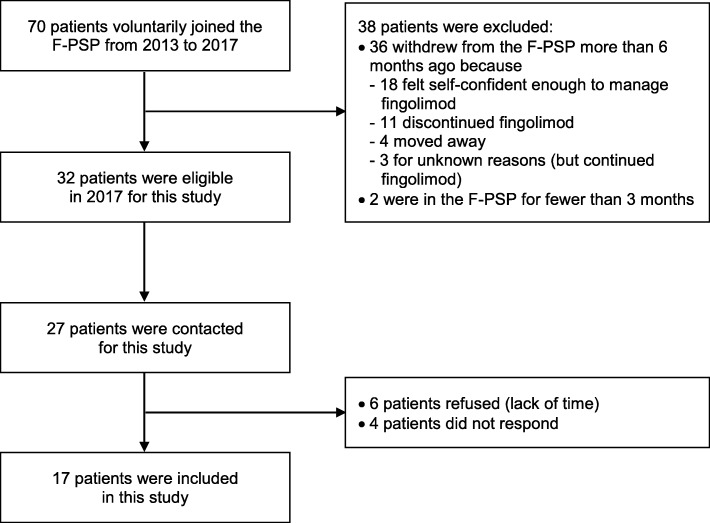
Patient flowchart. F-PSP: Fingolimod Patient Support Program

A sampling grid with predefined socio-demographic and clinical criteria was established to ensure maximal variation in representativeness, in order to explore the similarities and diversity in participants’ experiences, perceptions and beliefs related to the F-PSP. The criteria were age at F-PSP inclusion, gender, F-PSP setting (pharmacy type: Pharmacy of Unisanté or other), F-PSP duration and previous MS treatment experience. Patients were contacted as representatives of a maximum number of categories on the sampling grid. They were approached face-to-face or by phone contact. Recruitment continued until data saturation was reached (i.e., before the analysis, when both interviewers considered that no new information emerged from the interviews [24]) and a sufficient balance of the above-mentioned criteria was achieved. Thus, among the 32 eligible patients, 27 were approached, and 17 (63%) agreed to participate in the study (Fig. 1). All patients received an information document and signed a consent form prior to the interview.

### Data collection

Interviews were conducted in French by either AB or JD between March and July 2017. AB is a pharmacist PhD student trained in qualitative research. She proposed the F-PSP to most patients and conducted the F-PSP inclusion consultation, but she never delivered further F-PSP consultations to these patients. JD is an anthropologist and a qualitative research expert who had no prior contact with the patients.

A semistructured interview guide composed of open-ended questions (see Additional file 1) was used to conduct the interviews. The interview guide was developed based on our experience of the F-PSP and was discussed by our research group, which has strong experience with other medication adherence programs. The following main topics were addressed: overall program, pharmacist-led consultations, EM, interprofessional collaboration and patients’ opinions on the advantages and disadvantages of the F-PSP. Following the first interviews, the guide was slightly adapted by adding the following topics: location of the EM at home, drug intake graph discussed during the pharmacist-led consultations and exclusive or non-exclusive therapeutic relationship with an assigned pharmacist within the F-PSP. Interviews were audio-recorded and transcribed verbatim. Field notes were taken during the interviews.

Participants chose the interview location and received 20 CHF (in the form of a gift voucher) as compensation.

### Data analysis

The data were coded and analyzed using MAXQDA v.12 (VERBI Software) via open coding and thematic analysis [25]. Two interviews were double-coded by JD and AB. The coded transcripts were then compared for agreement, and the final codebook was applied to all the transcripts by AB (see Additional file 2). Each new code was discussed between JD and AB. JD cross-checked all the transcripts to ensure validity and rigor as well as consensus in the application of the codebook. Similar codes were then merged into subthemes and themes derived from the interview guide.

Representative quotations presented in the Results part and Additional file 3 were translated into English by a fluent English speaker, and both interviewers (AB and JD) double-checked the translation to make sure that the meaning was preserved.

## Results

Seventeen interviews were conducted (12 by AB and 5 by JD) between March and July 2017; they lasted from 36 to 77 min (median: 52 min). At the time of the interviews, the participants’ ages were between 19 and 58 years (median: 38 years), and they had participated in the F-PSP for 4.7 to 40.2 months (median: 29.2 months). Table 1 presents the participants’ characteristics. The proportions of the different categories were representative of the F-PSP population. At the time of the study, all participants were still in the F-PSP except one (SAT_10), who withdrew less than 3 months before the interview.

**Table 1:** Participants’ characteristics (*n* = 17)

		Number	Percentage
Gender	Male	7	41%
	Female	10	59%
Age at F-PSP’s inclusion	< 30 years	4	24%
	30–50 years	11	65%
	> 50 years	2	12%
Previous experience in MS treatment	Naïve^a^	11	65%
	Experimented	6	35%
F-PSP’s duration^b^	< 1 year	3	18%
	≥ 1 year	14	82%
F-PSP’s setting	Pharmacy of Unisanté	13	76%
	Other pharmacy	4	24%

F-PSP: Fingolimod Patient Support Program

^a^ patients who had never taken any MS treatment before the initiation of the F-PSP

^b^ at the beginning of the qualitative study (March 3, 2017)

The main themes were all derived from the interview guide, and no other new theme was identified. Those themes were 1. overall perception of the F-PSP, 2. perception of the pharmacist-led consultations, 3. perception of the tools, 4. reasons to participate or potentially withdraw, and 5. suggestions for improvements. Statements made by the participants are integrated into the body of the text. Additional file 3 lists supplementary representative quotations.

### Overall perception of the F-PSP

Many participants perceived the F-PSP as a reassuring support, especially regarding drug-related concerns, such as adverse events or interactions. A few noted that it enabled them to become involved in their treatment and feel empowered. While several participants perceived a positive impact of the program on their medication adherence (linked to either the tools—the EM or drug intake graph—or the pharmacist intervention), some believed that the program did not influence their medication use. Two said they would have taken their medication just as consistently without the program but admitted they would not have had the comfort and reassurance provided by the EM.

“*Coaching is actually reassuring for me. That is what tipped the scales. It gave me a positive opinion about the drug. I can call almost anytime, whether it is the physician or the pharmacy. They take note of potential adverse events, and they are listening. It is very reassuring for me. It turned my vision into something positive with the experience I had with this drug, I would say*.” SAT_03

“*It is nice to be involved in your treatment too. I think it allows you to get involved as a patient, take responsibility, and ask yourself questions regularly*.” SAT_02

A majority of participants described a more human, personalized and individualized approach with the F-PSP than with usual care in a pharmacy (without the program).

“*There is a very human follow-up. You do not feel like number 424. There is a more personal side that you would not get in a neighborhood pharmacy*.” SAT_06

Many participants agreed that pharmaceutical and medical care both have their own advantages and are different and complementary: the F-PSP was perceived as a global approach, and neurologist intervention was described as specifically disease-centered. Almost half of the participants found pharmacists to be more available during consultations than neurologists, whose time was limited by their schedule. Additionally, participants noted that pharmacy appointments were more frequent than neurology appointments, which reassured them.

“*We are all pulling the same rope to make sure I am healthy*.” SAT_01

“*Both approaches are balancing, I think. The pharmacy is maybe more open to the context and includes everything, whereas in neurology, it is more focused on the disease, and you do not take into account the related constraints.* (...). *Both approaches are two different things, though*.” SAT_17

In joining the program, most of the participants had to visit a pharmacy that was not their usual one. Nevertheless, most did not mind changing their pharmacy, and some recognized the following benefits: they could remain anonymous and thus guarantee discretion about their condition; the pharmacist consultations could be paired with their neurologist appointments (for participants visiting the Pharmacy of Unisanté); and the pharmacy was close to their workplace. Two participants admitted that they were initially annoyed that they had to visit another pharmacy, but neither would want to change now.

*“So at first it kind of annoyed me a little bit, let's say. Because my usual pharmacy is right there. The other one is a little further away. Now I'm used to it, it's okay, there's no problem, but at the time it was a problem. (...) Now, if I were asked to change, I would say no. No. I really don't want to change.”* SAT_07

The main F-PSP disadvantages raised by many participants were its time-consuming nature and the need to commute to the pharmacy (the F-PSP pharmacy was not necessarily in their neighborhood).

### Perception of the F-PSP pharmacist-led consultations

All the participants said they felt well and relaxed during the consultations and considered their duration and frequency to be adequate. They identified two kinds of support—medication-related and holistic—discussed the pharmacist’s attitude and reported some disadvantages of the consultations.

“*Well, if I have questions, I can ask them all, she* [the pharmacist] *takes time for everything. If you do not have any questions, it will not take more than a quarter of an hour. But if I need to talk a little more, I can take an hour or more to get my answers*.” SAT_07

#### Medication-related support

Regarding the medication-related support, the most important consultation topics mentioned by a majority of participants were related to drug safety, particularly adverse event monitoring. Several participants also valued the topic of drug interactions/contraindications, reminders about recommended medical tests and health outcome monitoring. Many participants appreciated the medication adherence support provided during the consultations for the advice on medication intake management (in daily life or while on holiday) and the drug intake graph.

“*On the other hand, it allowed me to establish a strategy, rigor and follow-up that would have been much more difficult to set up without this structure, these interviews*.” SAT_10

#### Holistic support

The participants reported receiving holistic support. Indeed, most participants appreciated addressing topics such as family, work, lifestyle habits, and their mood or emotional condition. Some participants compared these consultations to psychological support. A few valued being considered as a whole, underlining that behind the patient, there was a person.

“*There is not only the drug. There is someone behind it. You are more visible, I think, than the pillbox you receive. It goes further. Your concerns and worries are more taken into account*.” SAT_17

#### The pharmacist

All participants judged the pharmacist’s attitude positively regarding both their professional and human attributes. Nevertheless, a few participants mentioned having an unpleasant experience with a pharmacist they felt was too paternalistic, as they received reprimands for their behavior and lifestyle. Several participants underlined the pharmacists’ expertise related to the disease, considering them reliable sources of information. The majority appreciated the opportunity to ask questions and be counseled. Most participants valued pharmacists’ availability and their active and nonjudgmental listening. Participants received comfort and reassurance from a caring and empathic pharmacist when needed. Some also appreciated being congratulated on their drug intake.

“*I felt like I could really talk, they* [the pharmacists] *were not just robots taking notes because I was part of the program ( … ) there was an exchange*.” SAT_03

“*There was no judgment. You were considered a normal person, a person who was not sick*.” SAT_12

One participant highlighted the importance of the pharmacist’s attitude, which influences the patient-pharmacist relationship, and mentioned that the consultation topics were personal and sensitive and could be difficult to discuss.

Pharmacists were valued for being third-party interlocutors who were not family members, GPs or neurologists.

“*This allows you to talk to someone other than the physician and family. So, this allows you to talk to someone who knows about the disease. Basically, it is difficult to talk to the family about it. It was my feeling at the beginning. Here, you are talking to a neutral person you do not know. I thought it was very good. It helped me a lot at first*.” SAT_12

While at the Pharmacy of Unisanté, patients can be followed by different pharmacists; at other pharmacies, patients are assigned one pharmacist. Among the participants who used the Pharmacy of Unisanté, opinions were divided regarding having a dedicated pharmacist. Some reported that they were comfortable being seen by different pharmacists because this enabled them to obtain different opinions, and one explained that despite the diversity of pharmacists, the intervention remained consistent. Conversely, others would appreciate seeing the same pharmacist to strengthen the bond of trust, to avoid repeating themselves or to optimize the uniformity and continuity of care management. For the same reasons, participants attending other pharmacies unanimously emphasized the importance of having a single assigned pharmacist.

*“Anyway, I came across a pharmacy where things are going well. Because the person* [the pharmacist] *is always the same one, she’s assigned. [*Interviewer: *Is that important to you?] Yes, it is. No, I wouldn’t like to have a different person every time. That’s something that annoys me (...). I wouldn’t want to find myself in front of someone who doesn’t know, who has to reread everything to understand, well that’s not the point. It’s a matter of trust, of consideration and I’m very happy that it’s the same [person], that’s for sure.”* SAT_04.

#### Disadvantages of the pharmacist-led consultations

Although the consultations were appreciated overall, some participants considered them less useful over time, mainly due to their good tolerance of the drug. A minority said they were repetitive and boring or redundant with their medical consultations, and one participant judged them to be too frequent (at the time of the study, he was at the beginning of the program and was having monthly consultations).

### Perception of the tools

Participants identified two tools related to the F-PSP: the EM and the drug intake graph.

#### Electronic monitor

A majority of participants described automatic and routine drug intake. They appreciated the EM because it offers the reassuring possibility of checking their daily drug intake through the display on the cap screen. Indeed, several pointed out a risk of missing doses due to this routine (the habit of taking the drug daily may lead to doubts regarding whether it has been taken), and some noted the risk due to changes in the routine, for example, when on holiday or on weekends. For several participants, the EM helped prevent forgetting, especially because it flashes when it is not opened at the scheduled time but also simply because the object itself acts as a visual reminder.

“*If it* [the EM] *was not there, I might forget to take my pills more easily, simply put. Because if I just had these little tablets, it would be less obvious to me. The visible presence of this monitor is not stupid at all*.” SAT_04

Moreover, a number of participants explained that the EM enabled their family members, such as their children, partners or parents, to be involved. Relatives could check the EM, which was reassuring for both participants and relatives.

“*If my mother has any doubt, she checks* [the EM] *and knows that I have taken it* [the drug]*. She is reassured. It helps, let’s say*.” SAT_01

Several participants felt they benefited from using the EM due to the feedback regarding medication adherence obtained from the graph and the pharmacist intervention. Finally, it also helped some participants take their cotreatments by matching their intake with their EM use.

Interestingly, one participant fully appropriated the EM by naming and speaking to it, which facilitated her drug management. She used very strong words with martial connotations to describe it, considering the EM as an ally, a travel companion to support her in her fight against the disease. Otherwise, participants did not perceive any particular symbolism of the EM beyond its primary function as a drug dispenser.

“*I named it.* (...) *Its name is ‘PB-8’* ( … ) *in one of the last* [episodes of Star Wars] *there is a ‘BB-8’, the small white droid* ( … ) *so I named it ‘PB-8’ because PB is pill box in English.*” SAT_03

Most participants stored the EM in the kitchen, and half of these associated it with coffee (e.g., coffee machine, cup). Other locations were the bedroom, on the desk, on the dining table, and in the bathroom cabinet. Several participants highlighted the importance of storing it in a visible place to remind them to take the drug.

Although the majority of participants appreciated the EM, some considered it nonessential for their drug intake. The main reported disadvantage was its visual appearance, which was judged unsightly or bulky. The device reminded three participants of their disease, and one complained about occasional malfunctions. Moreover, most participants did not take it with them when they were traveling (preferring pocket doses) either because it was cumbersome or because they were afraid to lose it. Several participants did not perceive any disadvantages.

“*It is a little bit of a pity. This pillbox is not very nice, visually, I would say. We are in an era where everything is cool; I still find it very medical. Besides, it would be nice if there were a pocket size for holidays*.” SAT_15

#### Drug intake graph

All participants except one appreciated seeing and discussing the graph of their drug intake. It provided them with objective evidence of their intake over the last intervisit period, which reassured them. Almost half of the participants expressed self-satisfaction and pride when viewing their graph, as it showed the regularity of their intake. However, one participant mentioned that he would have experienced suboptimal adherence as a failure. Some reported challenging themselves to be adherent to have a “beautiful graph” at their next visit. Two participants mentioned the advantage of the graph for informing physicians of their adherence. Although some felt tracked or controlled at the beginning of the program, this feeling disappeared over time. Moreover, a few participants perceived it as a “positive control”, motivating them to take their treatment properly because their drug intake would be seen.

“*There is almost a rewarding side, ‘Ah, I took it on time!’. You can see I have been regular. It is like a small reward, you have physical evidence that you did your job well. So, it is quite cool*.” SAT_16

“*It is very interesting.* ( … )*. When she* [the pharmacist] *first showed me this graph, it was like I was at school; ‘I will get a good mark because I did it right’*. ( … ) *Besides, it was convenient to have this graph and it was a goal to reach for the next time: not having missed and always taking it* [the medicine] *at my usual time*.” SAT_03

Interestingly, a few participants used school-related vocabulary when discussing this topic: “It was like I was at school”, “have a good mark”, “a small sticker”, and “the good student.”

### Reasons to participate or potentially withdraw from the F-PSP

The main reason most participants joined the program was to be supported, and some reported wanting to be reassured or to feel secure. The support they sought was mainly related to medication adherence: to be helped to “not forget” and check their drug intake, especially with the EM, or to be coached to establish a rhythm for intake. Participants were also seeking support related to drug safety, either for adverse events or for the medical test reminders. Finally, some participants did not mention specific reasons other than general support for starting this new treatment. To help research was another reason mentioned by some.

Several participants associated their reason for participating in the F-PSP with their difficult situation (emotional - following a recent diagnosis delivery or following a relapse - or organizational) at treatment initiation. All but one of these were MS treatment-naïve and mentioned being “lost” or “in a panic” at that time. Drug-related concerns at initiation were raised by most participants and were linked to adverse events, medication adherence (based on cotreatments or previous MS treatment experience) and potential treatment ineffectiveness. Novelty was also a concern: new drug on the market, new galenic form, and new regimen were topics mentioned both by treatment-naïve and experienced participants.

“*I was a little lost, I did not know what to do and I found it nice to benefit from a follow-up, actually. It is true that when they explained the program to me, they told me that I would have a follow-up, that they would explain the appointments to me and I could also talk to someone about the little concerns about the disease and everything. So it is true that I at first, I thought it was very nice. It is what motivated me, actually*. ( … ) [Interviewer: *You said a follow-up, what does that mean to you?*] *Follow-up, it was about appointments with the dermatologist, ophthalmologist and everything that included the program with the intake of the Gilenya®, actually*.” SAT_12

Among the participants, only one had left the F-PSP at the time of the study. He withdrew because once the program helped him establish a medication intake routine, he no longer had time to devote to it. He also wanted to centralize his treatment supply in his usual pharmacy. The other participants raised four main hypothetical reasons for leaving the F-PSP: commute to the pharmacy (relocation, mobility issues), lack of time, costs incurred by the patient, and sufficient self-confidence to manage treatment alone.

### Suggestions for improvements to the F-PSP

The main suggestions for improvements to the F-PSP were linked to the following: 1. Accessibility: extending pharmacy timetables, disseminating the program to more community pharmacies, and ensuring synchronization between neurology and pharmacy appointments at the Pharmacy of Unisanté. 2. Pharmacist-led consultations: less frequent consultations and having an assigned pharmacist if requested. 3. EM: improving the aesthetics, designing a pocket format for holidays, and adding an alarm connected to the EM or a phone.

## Discussion

This qualitative study provides insights into the satisfaction, experiences and perceptions of patients supported by the F-PSP. The findings highlight the strengths and weaknesses of the F-PSP.

The program provides medication-related and holistic support. The medication-related support focuses on medication intake and safety. According to the participants’ statements, the monitoring of adverse events was valuable and reassuring. Notably, fingolimod has safety issues, and at treatment initiation, several participants had adverse-event-related concerns. Participants highly valued receiving information, education and counseling on their medication management and appreciated the opportunity to ask questions to a healthcare professional considered to be a reliable source of information. These findings emphasize patients’ need for information and reassurance, which suggests the potential for daily isolation in the face of treatment and/or disease. These needs have been observed among patients with other chronic conditions [26–31]. Tinelli et al. showed that patients expected a knowledgeable pharmacist to be able to answer their questions satisfactorily [32]. Additionally, the participants’ perceptions were consistent with the information-motivation-behavioral skills model [11] implemented by the pharmacists to promote patient adherence, whose components (knowledge, counseling and accountability) are known success factors of interventions enhancing medication adherence [13, 14].

According to the participants, holistic support was also important. By addressing non-medication-related topics in the consultations, the participants felt that they were treated as people rather than patients. Thus, the F-PSP can be considered person-centered and comprehensive. Therefore, this program contributes to the paradigm shift to person-centered care recommended by national and international policies [33–35]. Moreover, personal attention received from a pharmacist seems to positively influence patient satisfaction [36].

Pharmacists delivered the intervention through active listening, which was perceived to be a valuable skill. Pharmacists’ availability and competence were also appreciated, as in another support program [37]. The pharmacist’s attitude emerged as very important for building a trusting relationship, as this facilitated dialogue and encouraged the sharing of confidences. Participants felt uncomfortable with paternalistic attitudes. The literature suggests that a good relationship based on trust, respect and a supportive attitude positively influences clinical outcomes and medication adherence [38–40]. Additionally, patient-pharmacist communication has been identified as an enabling factor for the successful implementation of a pharmacist-led program [41] and a trusting patient-clinician relationship as beneficial for continuity of care [42]. Pharmacists being perceived as third-party interlocutors probably contributes to the establishment of this relationship. Notably, participants with assigned pharmacists highly valued these relationships. These findings are consistent with other studies emphasizing the importance of the patient-professional relationship from both the patient’s [27, 43] and pharmacist’s [44, 45] perspectives.

The program promotes patients’ medication adherence through a pharmacist intervention coupled with two tools: the EM and the drug intake graph. The EM was appreciated for promoting daily adherence. Participants appropriated the device by integrating it into their routine, and one took the appropriation as far as naming it and talking to it. Keeping the EM in a visible location seemed to facilitate drug intake. The drug storage locations in the domestic space appear important and to be related to their representation and the importance attached to them [46]. The possibility of involving relatives was an added benefit and reassured relatives and patients. Family is known to promote patients’ medication adherence [47–50]. These appropriation strategies, which had not been considered when designing the F-PSP, seem to consolidate its adoption and foster patient involvement in treatment management.

The graph can be perceived as a link between patients’ daily life and the program intervention. The drug intake graph was appreciated for providing an intervisit view of adherence, thereby rewarding and reassuring participants. It indirectly promoted their adherence by motivating them to challenge themselves to generate a “beautiful graph” or through the social desirability effect, as the patients knew that their drug intake was being monitored, which provided a “positive control”. Self-monitoring with tools is another key element for successfully enhancing medication adherence [13]. The information supplied by these two tools reinforced the pharmacist-led medication adherence support. The use of school-related vocabulary by participants when discussing the drug intake graph might reflect our participants’ desire to do things right and to show they fulfilled their role as a patient. However, further investigations should be undertaken to support these hypotheses.

The participants’ statements highlighted some weaknesses of the program related to the accessibility to the pharmacy, the EM and the pharmacist-led consultations. Based on these results and the participants’ suggestions, some measures for improvement are suggested for optimizing the program. Efforts must be made to enhance its accessibility, especially to increase its dissemination to more community pharmacies. The EM should be redesigned to make it more attractive. Patients who express a need for a dedicated pharmacist should be accommodated. Pharmacists should remain judgment-free and avoid paternalism.

The findings also suggested that participants’ treatment perceptions and emotional situation at treatment initiation influence their interest in the program. As these factors are personal and dynamic, no “standard profile” of patients likely to join the program can be established. The F-PSP should continue to be proposed to each new patient. The findings showed that the tools complement the pharmacist-led consultations; thus, it is important to give them equal emphasis.

Despite the overall participant satisfaction with the F-PSP, some features were not beneficial or were no longer beneficial after a certain time for some patients. However, at the time of this study, all participants but one were still participating in the F-PSP, indicating that the benefits derived from other aspects outweighed other considerations. Overall, the program seemed to be suitable for each participant but for different reasons, as each participant had their own needs. This PSP is intended to be a generic model transferable to other specialty drugs, diseases, populations and healthcare contexts. Indeed, since participants’ needs were not specifically related to MS, patients undergoing chronic treatments, such as HIV antiviral, oncological, diabetic, and antihypertensive regimens, etc., may benefit from a similar program. To respond to the findings of this study and national and international recommendations for healthcare systems, essential elements valuable for patients should be considered to develop pharmacist-led PSPs. First, pharmacists must be well trained in the management of the specific drug/disease to ensure that they have comprehensive clinical knowledge and competencies. Second, PSPs must rely on tools that provide daily support and objective feedback over a period of time. Third, pharmacists must have key human and communication competencies to establish a trusting relationship. To develop and maintain communication skills, pharmacists should attend ongoing training. Fourth, pharmacists must adopt a holistic and person-centered approach. Finally, a single pharmacist should be assigned to patients upon request.

This study has several limitations. First, participants who withdrew from the F-PSP for more than 6 months were not included, although they would have offered valuable additional insights. Nevertheless, this cutoff was chosen to limit recall bias. In addition, at the time of the study, all participants but one were still involved in the program and may therefore have had favorable opinions about it. However, only one other patient outside the F-PSP was eligible. Second, one of the authors worked at the Pharmacy of Unisanté (where the majority of interviewees participated the program) and participated in the enrollment of most of the patients in the F-PSP. However, this pharmacist researcher only conducted the F-PSP enrollment and never delivered F-PSP consultations. Moreover, for this study, she presented herself as a “researcher” instead of as a “pharmacist” and allowed the participants to choose their interview site to minimize potential interviewer bias. Third, as three authors contributed to the development of the F-PSP, the involvement of another researcher (an expert in qualitative research and social sciences) completely unrelated to the F-PSP process mitigated the risk of interpretation bias. Finally, three-quarters of participants visited one pharmacy site. However, this proportion was representative of the F-PSP population.

## Conclusions

This study demonstrated that participants were satisfied with the F-PSP, each for different reasons. They appreciated both the pharmacist-led consultations with medication-related and holistic support and the two tools. Consultations based on motivational techniques and the appropriation of tools supplied by PSPs foster patients’ involvement in their health (patients’ empowerment). This model seems to meet participants’ needs, to complement medical care, and to provide actual added value. From the patients’ points of view, pharmacists appear to contribute significantly to patient management, strengthening their legitimacy with respect to the provision of PSPs. These findings will also enable the optimization and scaling-up of the F-PSP and analogous services. To help other clinicians and service providers implement effective pharmacy support services, the perspectives of patients who withdrew from the F-PSP and of the pharmacists who delivered it should be studied.

## Supplementary information

**Additional file 1.**

**Additional file 2.**

**Additional file 3.**

## Data Availability

Raw qualitative datasets analyzed during the current study are not publicly available since consent for sharing data was not granted by participants; deidentified data may be available in French from the corresponding author on reasonable request.

## Abbreviations

CER-VD: Cantonal commission for the ethics of human research
EM: Electronic monitor
F-PSP: Fingolimod Patient Support Program
GP: General practitioner
MS: Multiple sclerosis
