# Supplementary material for: Satisfaction and experiences of patients taking fingolimod and involved in a pharmacy-based patient support program in Switzerland — a qualitative study
Source: BMC Health Serv Res. 2020 May 14;20:425. doi: 10.1186/s12913-020-05278-3 (PMC7227186; doi:10.1186/s12913-020-05278-3)
Supplement: Supplementary file 1 — Additional file 1. [file 12913_2020_5278_MOESM1_ESM.docx]

**If the patient is still enrolled in the program:**

| **Themes** | **Questions** | **Relaunch** |
| --- | --- | --- |
| Overall program experience | - What reasons motivated you to participate in this program? - What are the reasons that you are still engaged in this program? - According to you, what are the influences of this program on your medical care? | - Why did you agreed to participate in this program? - What vision did you have of the program before beginning the program? To what extent did the program change your vision? - What are the personal advantages of this program? - How is the program affecting your drug intake? - How is the program affecting your possible fears regarding adverse events? - What differences do you perceive in the pharmacy care versus usual care (without a support program)? |
| Consultation experience | - What do you think of the pharmacy consultations? - How do you perceive the usefulness of the pharmacy consultations? What is your experience with the usefulness of the pharmacy consultations? - What are the most important topics discussed in these consultations? | - How do you perceive the pharmacist’s attitude during these consultations? - Do you always have the same pharmacist conducting the consultations? What do you think of that? - How do you feel during the consultations? - What do you think of seeing and discussing the drug intake graph with the pharmacist during the consultations? |
| Electronic monitor (EM) experience | - What is your experience of having the EM at home? - How do you perceive the EM’s usefulness? What is your experience of the EM’s usefulness? - What does the EM represent for you? | - What do you think of the EM? - What are the advantages or disadvantages of the EM? - Where do you store the EM? Why? |
| Interprofessional collaboration experience | - According to you, what is the role of your neurologist, nurse and pharmacist in this program? - How do you perceive the collaboration between the pharmacy and the medical team? |  |
| Advantages / disadvantages | - According to you, what are the advantages/disadvantages of this program? - If you could, what improvements would you make to this program? - What are the reasons that could cause you to withdraw from the program? - What is your opinion about the fact that the program is implemented in a pharmacy other than your usual pharmacy? | - What do you think of the frequency of the pharmacy consultations? - What do you think of the length of the pharmacy consultations? |
|  | - Would you recommend this program to other patients? | Why? |

EM: Electronic monitor

**If the patient is no longer enrolled in the program (same questions in past tense except the italics questions, which were different):**

| **Themes** | **Questions** | **Relaunch** |
| --- | --- | --- |
| Overall program experience | - What reasons motivated you to participate in this program? - *What were the reasons that you continued this program during…..months/years?* - *What were the reasons that made you quit this program?* - According to you, what were the influences of this program on your medical care? | - Why did you agree to participate in this program? - What vision did you have of the program before beginning the program? To what extent did the program change your vision? - What were the personal advantages of this program? - How did the program affect your drug intake? - How did the program affect your possible fears regarding adverse events? - What differences did you perceive in the pharmacy care versus usual care (without a support program)? |
| Interview experience | - What did you think of the pharmacy interviews? - How did you perceive the usefulness of the pharmacy interviews? What was your experience with the usefulness of the pharmacy interviews? - What were the most important topics discussed in these interviews? | - How did you perceive the pharmacist’s attitude during these interviews? - Did you always have the same pharmacist conducting the interviews? What did you think of that? - How did you feel during the interviews? - What did you think of seeing and discussing the drug intake graph with the pharmacist during the interviews? |
| Electronic monitor (EM) experience | - What was your experience of having the EM at home? - How did you perceive the EM’s usefulness? What was your experience of the EM’s usefulness? - What did the EM represent for you? | - What did you think of the EM? - What were the advantages or disadvantages of the EM? - Where did you store the EM? Why? |
| Interprofessional collaboration experience | - According to you, what was the purpose of your neurologist, nurse and pharmacist in this program? - How did you perceive the collaboration between the pharmacy and the medical team? |  |
| Advantages / disadvantages | - According to you, what are the advantages/disadvantages of this program? - If you could, what improvements would you make to this program? - What is your opinion about the fact that the program was implemented in a pharmacy other than your usual pharmacy? | - What did you think of the frequency of the pharmacy interviews? - What did you think of the length of the pharmacy interviews? |
|  | - Would you recommend this program to other patients? | Why? |

EM: Electronic monitor
