# Supplementary material for: Satisfaction and experiences of patients taking fingolimod and involved in a pharmacy-based patient support program in Switzerland — a qualitative study
Source: BMC Health Serv Res. 2020 May 14;20:425. doi: 10.1186/s12913-020-05278-3 (PMC7227186; doi:10.1186/s12913-020-05278-3)
Supplement: Supplementary file 2 — Additional file 2. [file 12913_2020_5278_MOESM2_ESM.docx]

| Main themes | Subthemes | Codes |
| --- | --- | --- |
| Overall perception of the F-PSP | Support | Reassuring |
|  |  | Empowering |
|  |  | Influence on medication adherence |
|  |  | More personalized than usual care in the pharmacy |
|  |  | Complementarity with medical care |
|  | F-PSP pharmacy versus usual pharmacy | No inconvenience to change the pharmacy |
|  |  | Anonymity / Discretion |
|  |  | Pairing pharmacist and neurologist appointments |
|  |  | Proximity with workplace |
|  | Interprofessional collaboration | Perceived |
|  |  | Not perceived |
|  | Disadvantages | Time-consuming |
|  |  | Commuting to the pharmacy |
| Perception of the pharmacist-led consultations | Practical aspects | Duration |
|  |  | Flexibility |
|  | Medication-related support | Drug safety related |
|  |  | Medication adherence related |
|  | Holistic support | Person as a whole |
|  |  | Psychological support |
|  | Pharmacist | Expert / Reliable source of information |
|  |  | Third-party interlocutor |
|  |  | Pharmacist's attitude |
|  |  | Importance of pharmacist's attitude |
|  |  | Importance of an assigned pharmacist |
|  |  | Unpleasant experiences |
|  | Drawbacks | Less useful over time |
|  |  | Repetitive / Boring / Redundant with medical appointments |
|  |  | Too frequent |
| Perception of the tools | Electronic monitor | Daily checking of fingolimod intake |
|  |  | Reminder |
|  |  | Involvement of relatives |
|  |  | Relation / Appropriation |
|  |  | Location |
|  |  | Disadvantages |
|  | Drug intake graph | Feedback |
|  |  | Control |
| Reasons to participate or potentially withdraw from the F-PSP | Reasons to participate | Support |
|  |  | Research |
|  |  | Related to the situation at treatment initiation |
|  | Reasons to withdraw | Routine of medication intake established |
|  |  | No longer time to devote to it |
|  | Reasons to potentially withdraw | Commute to the pharmacy |
|  |  | Lack of time |
|  |  | Costs supported by patients |
|  |  | Sufficient self-confidence to manage treatment alone |
| Suggestions for improvements to the F-PSP | Accessibility | Pharmacy timetables extension |
|  |  | Disseminating the program to more pharmacies |
|  |  | Pairing pharmacist and neurologist appointments |
|  | Consultations with pharmacist | Assigned pharmacist |
|  |  | Frequency |
|  | Electronic monitor | Aesthetics |
|  |  | Pocket format |
|  |  | Alarm |

F-PSP: Fingolimod Patient Support Program
