# Supplementary material for: Satisfaction and experiences of patients taking fingolimod and involved in a pharmacy-based patient support program in Switzerland — a qualitative study
Source: BMC Health Serv Res. 2020 May 14;20:425. doi: 10.1186/s12913-020-05278-3 (PMC7227186; doi:10.1186/s12913-020-05278-3)
Supplement: Supplementary file 3 — Additional file 3. [file 12913_2020_5278_MOESM3_ESM.docx]

**Table 1** Overall perception of the Fingolimod Patient Support Program (F-PSP)

| Subthemes | Participant quotations |
| --- | --- |
| Support | “*It has a good influence on drug intake. You do not feel watched, but you feel there is someone behind you if you ever forget or to talk to anytime if there is a problem. It is easier*.” SAT_17  “*If I did not follow this program, I would do like everything else, I would go to the pharmacy with the prescription, they would give me the medication, I would come back home, and if you have doubts, you stay with your doubts*.” SAT_07  “*Well, I think I would take* [the medicine] *too* [without the program]. *But I would be insecure if I did not have the pillbox.*” SAT_15  “*I think it* [the program] *is more global. I think it includes the disease, but also external factors, such as family and feeling. This is less present with the neurologist.*” SAT_12 |
| Interprofessional collaboration | “*So, there may be a collaboration, but nowadays, I do not feel it when I talk to one or the other*.” SAT_04 |
| Disadvantages | “*The disadvantage is that you have to insert this in your diary*.” SAT_05  “*If it were near my house, I would not mind. But it is more about the travel [to the pharmacy] and the time it takes for everything*.” SAT_03 |

**Table 2** Perception of the F-PSP pharmacist-led consultations

| Subthemes | Participant quotations |
| --- | --- |
| Medication-related support | “[Interviewer: *During the interviews, which were, in your opinion, the most important topics that you discussed?*] *It was really adverse events from the drug. For me, it was the most important*.” SAT_03  “*The most important topic was probably the possible contraindications*.” SAT_13  “*Rather, it was advice on monitoring, on the correct drug intake*.” SAT_12  “[Interviewer: what is important for you to discuss in these consultations?] *How the treatment is going, whether everything is going well or whether there is a doubt*.” SAT_17  “*We talked a lot about scheduling appointments* [of the recommended medical tests*]. For me it was, it was a topic that seemed to be a problem at the beginning*.” SAT_09 |
| Holistic support | “*So it is true that you are talking to a neutral person, whom you do not know after all, then I found it very well. Really, it helped me a lot at first.* [Interviewer: Yeah, to have someone external...] *External, yes, who knows nothing about you but to whom you can tell your problems, your* worries *and your feelings. I found it really nice*.” SAT_12 |
| The pharmacist | “*An available person can help to talk about it. Simply put! If you do not really want to come to the appointment because you are tired or stressed, the pharmacist is still welcoming. As a result, it is going well*.” SAT_04  “*It was reassuring to know that she* [the pharmacist] *had taken note. (…) I can call if there is a change or anything else. It is comforting to know that someone is listening and taking note*.” SAT_03  “*Well, what worried me at first was the adverse events.* [Interviewer: How did she reassure you?] *Every time, when she was listening to what worried me*.” SAT_15  “*Yeah, yeah, I like it. First, because I get congratulations for taking my medication properly. I am someone like that; I follow the “Weight Watchers” program because I love to be told “bravo!”*.” SAT_15 |

**Table 3** Perception of the tools

| Subthemes | Participant quotations |
| --- | --- |
| Electronic monitor | “*The advantage is that it is a permanent guarantee that you have taken* [the medicine] *or should take it*.” SAT_15  “*It allows my wife to control my medication intake too. So this is a double check after all*.” SAT_12  “*It is a little contradictory, because it* [the electronic monitor] *is cumbersome, but at the same time it is an eye-catching object, that you see and that allows the drug intake.*” SAT_12  “*It* [the electronic monitor] *is there to fight the disease and slow it down at least. So I see it as an ally, my traveling companion in MS. It is there to assist me, to fight the disease I would say. So, I must not forget*.” SAT_03  “*The disadvantage is its size*.” SAT_10 |
| Drug intake graph | “*So for me it* [the graph] *was a source of pride. Because I was regular, and it made me happy to watch it*.” SAT_08  “*There is almost a rewarding side, ‘Ah, I took it on time!’. You can see I have been regular. It is like a small reward, you have physical evidence that you did your job well. So, it is quite cool.”* SAT_16  “*It is very interesting. (…). When she* [the pharmacist] *first showed me this graph, it was like I was at school; ‘I will get a good grade because I did it right’. (…) Besides, it was convenient to have this graph and it was a goal to reach for the next time: not having missed and always taking it* [the medicine] *at my usual time*.” SAT_03  “*I have never been bothered by the feeling of control that makes you take* it [the drug] *all the time*.” SAT_12 |

**Table 4** Reasons to participate or potentially withdraw from the F-PSP

| Subthemes | Participant quotations |
| --- | --- |
| Support | “*I think it is useful to have someone to talk to about the experience and possible adverse events you may feel. It is a support*.” SAT_03  “*It was the fact of having a follow-up too and what was especially important for me was to check that I take it every day*.” SAT_03  “*It is just to have an obligation and to help me keep up with the pace of intakes. Well, in some ways, when you are compelled to report intake, you respect the rules*.” SAT_13 |
| Research | “*On the other hand, it allows a better understanding of some mechanisms, which will help research and therefore care itself*.” SAT_05 |
| Situation at treatment initiation | “*It was quite new for me, you still are on the first year, and this year is very complicated. Two months after* [the diagnosis]*, it was ‘panic on board’. It was the first drug for multiple sclerosis, and it was the first drug I was going to take for a long time*.” SAT_07  “*The circumstances were a little special because I started Gilenya^®^ treatment and in fact, I just had a multiple sclerosis relapse, so I was not feeling very well*.” SAT_02  “*There was drug intake, rehabilitation, there were many things at the same time that made it impossible for me to think of everything.*” SAT_10  “*As everything falls on you, it is true that to feel accompanied is a good point. It motivates. So that is mostly that, to feel coached in something you do not know at all, and that can be a little bit frightening*.” SAT_10 |
